# Identification of dominant peptide epitopes and antibody response to the spike protein of SARS-CoV-2

**DOI:** 10.3389/fcimb.2025.1670219

**Published:** 2025-10-15

**Authors:** Xianyan Zhang, Peipei Xu, Ting Xiao, Xinlu Wang, Xuemin Guo

**Affiliations:** ^1^ Clinical Laboratory Center, Meizhou People’s Hospital, Meizhou, Guangdong, China; ^2^ Guangdong Engineering Technological Research Center of Clinical Molecular Diagnosis and Antibody Drugs, Meizhou, Guangdong, China; ^3^ State Key Laboratory of Biomacromolecules, Institute of Biophysics, Chinese Academy of Sciences, Beijing, China

**Keywords:** SARS-CoV-2, spike protein, dominant peptide epitopes, inactivated vaccine, antibody response

## Abstract

**Objective:**

Multiple antigenic epitopes are present in the spike (S) protein of severe acute respiratory syndrome coronavirus 2 (SARS-CoV-2), but they exhibit significant variation in immunogenicity and reactogenicity. We aimed to investigate the reactivity of different SARS-CoV-2 antigenic epitopes with serum antibodies from patients with COVID-19 and vaccinated individuals to characterize specific antibody responses.

**Methods:**

Linear B-cell peptides derived from the SARS-CoV-2 S protein were screened using the Immune Epitope Database (IEDB). These linear peptides were coupled to bovine serum albumin (BSA), and blot hybridization was used to investigate antigen–antibody reactions among the peptides, commercial anti-S protein polyclonal antibodies, and sera derived from vaccinated individuals and those with confirmed COVID-19. Sera from unvaccinated patients and BSA served as controls.

**Results:**

Six linear B-cell peptide epitopes were identified from the IEDB and designated as P1–P6. Among these, P6 (aa 809–826) demonstrated stronger reactivity with rabbit anti-SARS-CoV-2 S polyclonal antibodies than the others. Antibodies against P2 (aa 553–570) were detected in the early stages of infection in four patients with confirmed COVID-19, and two of them developed antibodies against P5 (aa 601–640) later during the middle or late stages. Antibody responses against P1 (aa 209–226), P3 (aa 769–786), P4 (aa 287–317), and P6 were either extremely weak or undetected in patients with confirmed COVID-19. Sera from 45 individuals immunized with the inactivated vaccine were tested for reactivity against the six peptides. Antibody signals against P2, P5, and P6 were detected either individually or in combination, with P2 showing the highest frequency, followed by P6 and P5. Antibodies against P1, P3, and P4 were not detected. Six predicted B-cell dominant peptides exhibited distinct immunogenicity and reactogenicity, with P2 (aa 553–570) eliciting the strongest response. Antibody profiles against S protein epitopes differed between individuals infected with SARS-CoV-2 and those vaccinated with inactivated vaccines.

**Conclusion:**

The peptide combination of P2, P5, and P6 shows potential for the development of peptide-based target antigens for SARS-CoV-2 serological antibody diagnostic reagents, and reveals the response patterns of antibodies against SARS-CoV-2.

## 1 Introduction

The 2019 coronavirus disease (COVID-19) is a global pandemic caused by severe acute respiratory syndrome coronavirus 2 (SARS-CoV-2). The genome of SARS-CoV-2 encodes four structural proteins—spike (S), envelope (E), membrane (M), and nucleocapsid (N)—along with 16 nonstructural proteins (nsp1–nsp16) and other accessory proteins (Krafcikova et al., 2020). The spike protein contains two subunits: the S1 subunit recognizes angiotensin-converting enzyme 2 receptors on the surface of host cells, and the S2 subunit mediates fusion of the viral membrane with the host membrane (Krafcikova et al., 2020).

An immune response is triggered following SARS-CoV-2 infection in humans. Different subtypes of anti-SARS-CoV-2 antibodies, including IgM, IgG, and IgA, can be detected in the serum after infection (Stoddard et al., 2021). The dynamics of specific antibody responses elicited by SARS-CoV-2 infection generally follow the characteristic pattern of antibody production following viral exposure (Arkhipova-Jenkins et al., 2021; Ong et al., 2021). IgG plays a major role in antiviral immunity and serves as a key marker for monitoring and evaluating immune status after vaccination. In addition, IgG is considered a significant auxiliary diagnostic indicator of SARS-CoV-2 infection, with a detection rate of 95% in adults with confirmed COVID-19 (Arkhipova-Jenkins et al., 2021). B-cell antigenic epitopes derived from SARS-CoV-2 stimulate the immune system to produce antibodies, among which neutralizing antibodies bind to specific neutralizing antigenic epitopes to block viral invasion and replication. B-cell epitopes are categorized as either conformational or linear. Conformational epitopes consist of discontinuous amino acid residues that are spatially adjacent in the three-dimensional structure of the protein. In contrast, linear epitopes, composed of continuous amino acid sequences, are easy to synthesize and validate, making them particularly useful in immunological research and vaccine development (Negi and Braun, 2017; Cai et al., 2021).

Different immunodominant B-cell linear peptides from SARS-CoV-2 have been screened using multiple methods and serologically validated in several studies. The S protein is the main target of such screening due to its key role in the binding and entry of the virus into host cells. Antibody profiles against the S protein have been studied in patients with COVID-19 and individuals vaccinated with either inactivated SARS-CoV-2 vaccines or mRNA vaccines (Li et al., 2020; Poh et al., 2020; Fraley et al., 2021; Ma et al., 2021). Poh et al. identified two dominant epitopes, S14P5 and S21P2, on the SARS-CoV-2 S protein using overlapping linear B-cell peptide pool (Poh et al., 2020). The main binding regions of antibodies in the serum of patients with COVID-19 were analyzed using peptide microarrays, revealing that specific antibodies predominantly react with epitopes in three regions: the S-CTD (aa 553–684), the fusion peptide and S2’ cleavage sites (aa 764–829), and aa 1148–1159 region (Li et al., 2020). Interestingly, the antibody profiles of sera from individuals vaccinated with either the inactivated vaccine BBIBP-CorV or the mRNA vaccine BNT162b2 exhibited partially overlapping regions within the antibody hotspot recognition areas (the S-NTD and RBD regions) (Fraley et al., 2021; Ma et al., 2021). Additionally, in the sera of volunteers who received two doses of BBIBP-CorV, peptides S1–5 (aa 25–36) and S2–22 (aa 812–823) may serve as potential biomarkers for assessing the effectiveness of neutralizing antibodies after vaccination (Ma et al., 2021). These findings highlight multiple antigenic epitopes on the S protein with significant variation in immunogenicity and reactogenicity. Antibody profiles against the S protein have been studied in both patients with COVID-19 and recipients of SARS-CoV-2 inactivated vaccines. However, previous studies have been limited by small sample sizes and strain types.

Serological IgG antibody testing is a key diagnostic modality for assessing previous infections, post-vaccination antibody levels, and understanding disease progression, offering the advantages of simplicity and rapid turnaround. Different antigenic epitopes vary in their immunogenicity and reactogenicity. The selection of suitable antigenic epitopes is a critical step in developing serological antibody immunoassay reagents. Serum antibodies against common cold-associated human coronaviruses (HCoVs) show high seroprevalence in the population (Zhou et al., 2013; Mulabbi et al., 2021). Most SARS-CoV-2 serological antibody kits target the S protein, RBD region, or N protein; however, these kits may cross-react with antibodies against common cold-associated HCoVs, resulting in false positives (Liu et al., 2020; Lou et al., 2020; Zhao et al., 2020; Tso et al., 2021). The production and use of natural antigens in diagnostic reagents are further hampered by SARS-CoV-2 variants, leading to increased production and purification costs (Lorenzo et al., 2021). Using immunodominant synthetic peptides as target antigens can alleviate many of these issues (Skwarczynski and Toth, 2016).

In this study, six B-cell epitopes from the S protein were predicted using a public Immune Epitope Database (IEDB). Serum samples from unvaccinated, vaccinated healthy volunteers, and patients with confirmed COVID-19 were collected and tested for antibodies. Our study may contribute to a better understanding of antibody production against different epitopes has different characteristics, and provide experimental data for the screening of antigenic peptides for serological diagnosis.

## 2 Materials and methods

### 2.1 Patients and sample collection

Sixty-nine individuals were recruited for this study, including 45 volunteers who had received two doses of the inactivated BBIBP-CorV vaccine (Beijing Institute of Biological Products Co, Ltd., Beijing, China), 20 unvaccinated volunteers with no history of SARS-CoV-2 infection, and four patients with confirmed COVID-19. Serum from four patients with confirmed COVID-19 (c1–c4) was collected at different stages of hospitalization, including two with severe illness (c1 and c2) and two with mild illness (c3 and c4). The characteristics of the study participants are provided in **Supplementary Table 1**. The collected sera were categorized into three groups: vaccinated, unvaccinated, and confirmed COVID-19. Blood serum was collected and stored at –80°C. Informed consent was obtained from all participants in accordance with the Declaration of Helsinki. This study was approved by the Ethics Committee of Meizhou People’s Hospital (approval number: MR-44-21-014309).

### 2.2 SARS-CoV-2 S protein B cell peptides screening

Linear B-cell peptides on the SARS-CoV-2 Wuhan-Hu-1 S protein (reference sequence, GenBank: YP009724390.1) were predicted using the online search function of IEDB (http://www.iedb.org/). After accessing the IEDB online webpage, the BepiPred 2.0 tool for predicting B-cell linear epitope was used to identify antibody-binding epitopes on the S protein. Linear peptides with at least five consecutive amino acids, each having an epitope probability score ≥ 0.5, were screened for further validation (Jespersen et al., 2017). The selected peptides were coupled to bovine serum albumin (BSA) and synthesized by Sangon Biotech (Shanghai, China) Co., Ltd. The structural data of the immunogenic peptide were visualized on the SARS-CoV-2 S protein (PDB ID: 6VSB) using PyMOL (Schrödinger, version 2.5). S protein sequences of various epidemic variants—Alpha (B 1.1.7; GenBank: QUE20269.1), Beta (B.1.351; GenBank: QVI03430.1), Gamma (P.1; GenBank: QXF22408.1), Delta (B.1.617.2; GenBank: QWE90280.1), Omicron (BA.1.1.529; GenBank: UHB27505.1), Omicron (BA.2; GenBank: UOZ41747.1), Omicron (BA.3; GenBank: WKF16558.1), Omicron (BA.4; GenBank: UPP14409.1), Omicron (BA.5; GenBank: UOZ45804.1), Omicron (XBB.1.5; GenBank: WBG44565.1), and Omicron (NB.1.8.1; GenBank: XRO60489.1)—were obtained from the NCBI database. Amino acid sequences of the six peptides and various epidemic variants were aligned using Clustal Omega (https://www.ebi.ac.uk/jdispatcher/msa/clustalo) and visualized with Jalview 2.11.4.0.

### 2.3 SARS-CoV-2 serum antibody test

Serum samples were collected from both the unvaccinated and vaccinated groups. SARS-CoV-2 S protein-specific IgM and IgG antibodies were detected using the 2019-nCoV IgM/IgG Antibody Detection Kit (chemiluminescent immunoassay; Maccura, Sichuan, China). The assay was performed according to the manufacturer’s instructions. A S/CO ratio≥1 was considered positive.

### 2.4 Western blot and silver staining

Western blotting was performed using the Simple Western system (ProteinSimple, San Jose, CA, USA), following the manufacturer’s instructions. IgG-free BSA (001-000-162, Jackson ImmunoResearch, West Grove, PA, USA) and epitope-BSA conjugates (50 ng/μL) were used as antigens and dispensed into designated wells of a 25-well plate (SM-W004, ProteinSimple). Primary antibodies included rabbit polyclonal anti-SARS-CoV-2 spike antibody (1:60, 40589-T62, Sino Biological, Beijing, China) and rabbit polyclonal anti-SARS-CoV spike antibody (1:60, 40150-T62, Sino Biological). A horseradish peroxidase (HRP)-conjugated anti-rabbit IgG secondary antibody (042-206, ProteinSimple) was also used. Images were analyzed using Compass software (v4.1.0, ProteinSimple). In addition, we separated epitope-BSA conjugates using SDS-PAGE and stained them using a silver staining kit (Beyotime Biotechnology, Shanghai, China).

### 2.5 Dot blot analysis

Fifty nanograms of recombinant SARS-CoV-2 S protein (40589-V08B1, Sino Biological, Beijing, China), IgG-free BSA, and peptides-BSA conjugates were spotted onto polyvinylidene fluoride membranes. After air drying, membranes were blocked with 10% skimmed milk for 1 h, then incubated overnight with sera from the unvaccinated, vaccinated, and confirmed COVID-19 groups, diluted to 1:5 in 10% skimmed milk. Membranes were washed in tris-buffered saline with tween-20, incubated with HRP-conjugated goat anti-human IgG (1:10000; 109-035-088, Jackson ImmunoResearch) for 1 h at room temperature. Dot blot signals were visualized using an ultrasensitive chemiluminescent solution (4AW011-20, 4A Biotech, Beijing, China), following the manufacturer’s instructions.

### 2.6 Data analysis

Data were analyzed using SPSS 23.0 software (IBM, Armonk, NY, USA), plotted in GraphPad Prism version 8 (GraphPad Software, San Diego, CA, USA). Final graphs were assembled in Adobe Illustrator CC2017 (Adobe Systems, San Jose, CA, USA).

## 3 Results

### 3.1 Dominant linear B-cell epitopes from SARS-CoV-2

Six linear B-cell peptides, each consisting of at least five consecutive amino acids with an epitope probability score ≥ 0.5, were identified from the SARS-CoV-2 S protein using the online BepiPred 2.0 prediction tool available on IEDB. These peptides were designated as P1 to P6. Their amino acid sequences and positions are shown in **Table 1**. After synthesis and conjugation with BSA, the peptides were used in subsequent assays.

**Table 1:** Identification of six linear B-cell epitopes from the SARS-CoV-2 S protein based on IEDB.

Peptide name	Sequence	Amino acid position
P1	PINLVRDLPQGFSALEPL	209–226
P2	TESNKKFLPFQQFGRDIA	553–570
P3	GIAVEQDKNTQEVFAQVK	769–786
P4	DAVDCALDPLSETKCTLKSFTVEKGIYQTSN	287–317
P5	GTNTSNQVAVLYQDVNCTEVPVAIHADQLTPTWRVYSTGS	601–640
P6	PSKPSKRSFIEDLLFNKV	809–826

### 3.2 Reactivity of peptides with commercial anti-S protein polyclonal antibody

**Figure 1:**
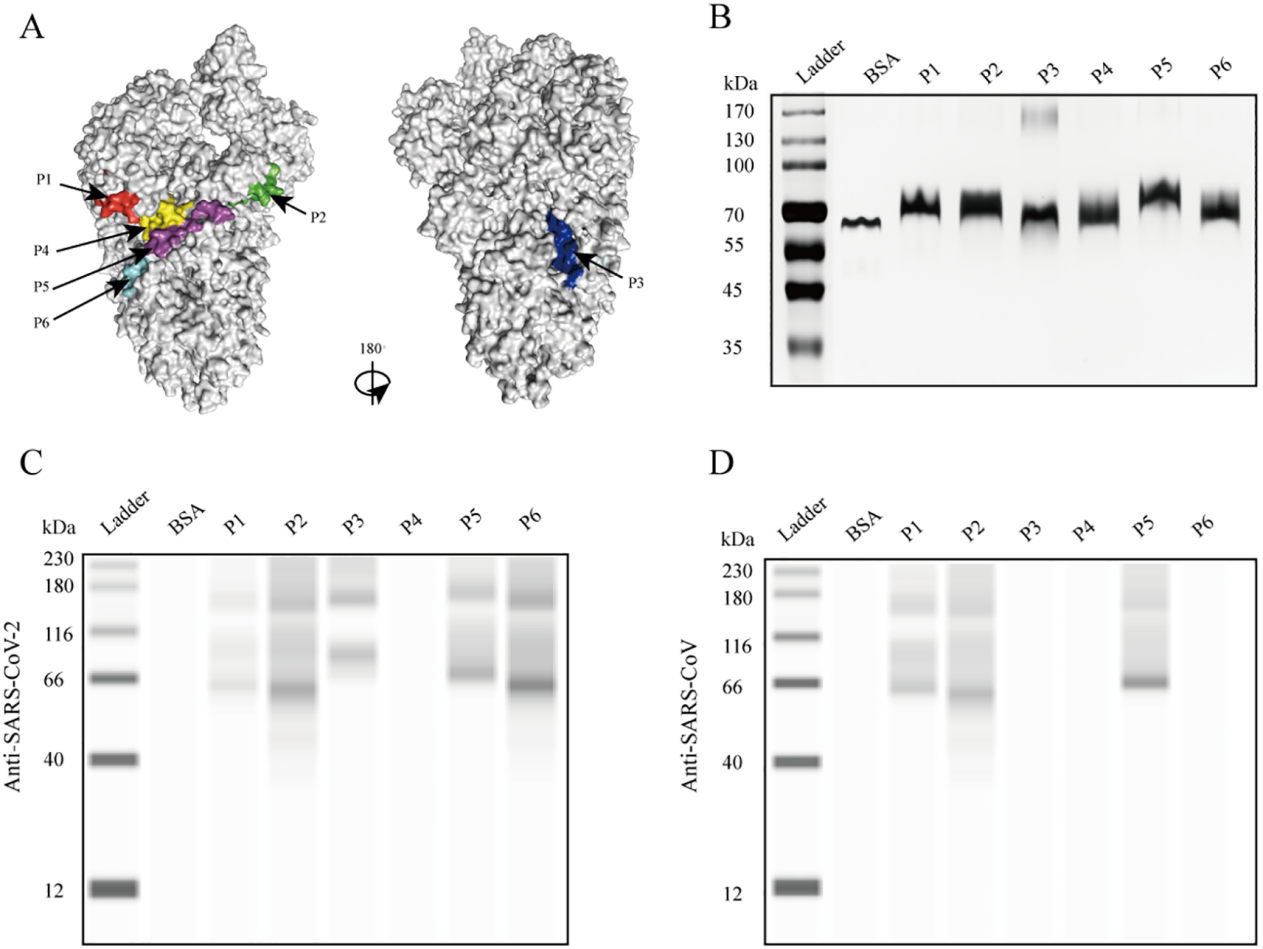
Reaction of the six peptides reacted with commercial anti-S protein polyclonal antibodies. **(A)** Localization of identified epitopes mapped on the SARS-CoV-2 S protein structure (PDB ID: 6VSB). The 3D structure is shown from different angles, with the positions of B-cell epitopes indicated as follows: red (P1), green (P2), blue (P3), yellow (P4), purple (P5), and cyan (P6). **(B)** SDS-PAGE separation of the peptides, visualized by silver staining. **(C)** Immunoblot showing peptide reactivity with anti-SARS-CoV-2 S protein polyclonal antibody. **(D)** Immunoblot showing peptide reactivity with anti-SARS-CoV S protein polyclonal antibody.

The dominant epitopes were mapped onto the structure of the S protein (PDB ID: 6VSB). Five peptides were located on the surface of the S protein except for P3, which was located in a groove on the surface of the protein (**Figure 1A**). The reactivity of the six dominant peptides with commercial anti-SARS-CoV-2 and anti-SARS-CoV S protein polyclonal antibody was analyzed using Simple Western. Equal protein loading of the six peptides was confirmed by SDS-PAGE and visualized with silver staining (**Figure 1B**). P1, P2, P5, and P6 reacted with the commercial anti-SARS-CoV-2 S protein antibody (**Figure 1C**). However, reactions with the commercial anti-SARS-CoV S protein antibody showed that P1, P2, and P5 were reactive (**Figure 1D**). No cross-reactivity was observed for P4 with either of the two commercial anti-S protein antibodies (**Figures 1C, D**). Compared to the anti-SARS-CoV S protein antibody, P6 showed strong reactivity only with the anti-SARS-CoV-2 S protein antibody (**Figures 1C, D**).

### 3.3 Reactivity of peptides with serum IgG antibody from patients with confirmed COVID-19

**Figure 2:**
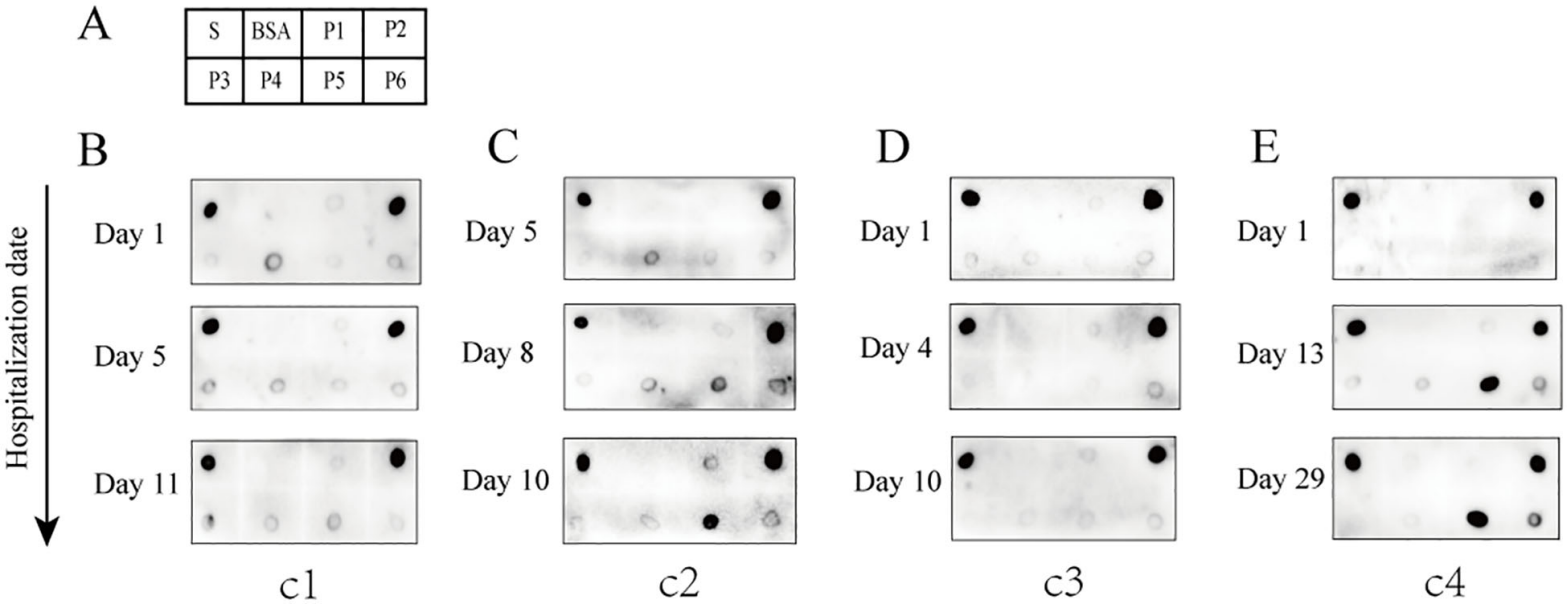
Reactivity of the six peptides with serum IgG antibodies from patients with confirmed COVID-19 at different disease stages. **(A)** Dot blot layout showing the peptide loading sequence. The S protein was used as a positive control, and bovine serum albumin (BSA) as a negative control. **(B–E)** Dot blot assay results are shown for patients c1 to c4. Patients c1 and c2 had severe disease; patients c3 and c4 had mild disease.

To assess the presence of antibodies targeting various epitopes of the S protein in patients with COVID-19, we evaluated the reactivity of the six peptides with sera from four patients with confirmed COVID-19 at different stages of hospitalization using dot blot assays. In the early stages of infection, a strong anti-P2 antibody signal was detected in the sera of all four patients and persisted throughout hospitalization (**Figures 2B–E**). Antibody signals against P5 progressively appeared in the sera of two patients as the disease progressed, while the other two patients showed no such response (**Figures 2C, E**). Antibody signals for P1, P2, P4, and P6 were undetectable or weak. These findings indicate that different antigenic epitopes have significantly varied immunogenicity and reactivity. Among the six peptides, P2 appeared to be the most immunogenic and reactive, with antibodies against it appearing earliest. In addition, antibody responses targeting the P5 epitope differed among patients.

### 3.4 Reactivity of peptides with serum IgG antibody from vaccinated volunteers

**Figure 3:**
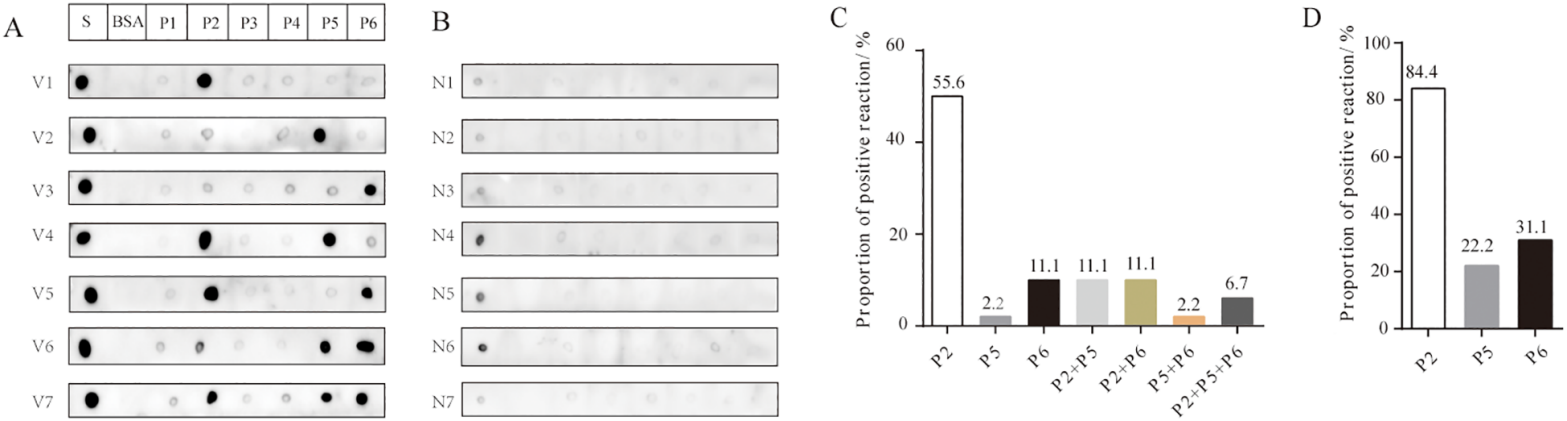
Reactivity of peptides with serum IgG antibodies from vaccinated and unvaccinated individuals. **(A)** Representative dot blot images showing the reactivity of the six peptides with serum IgG antibodies from vaccinated individuals. The S protein and BSA served as positive and negative controls, respectively. V1–V7 represent sera from volunteers after two doses of the vaccine. **(B)** Representative dot blot images showing peptide reactivity with sera from unvaccinated individuals (N1–N7). **(C)** The types of IgG antibodies bound to peptides and their positive reaction rates in each serum sample from the vaccination group. **(D)** The total positive reaction rates of serum IgG antibodies to P2, P5, and P6 in the vaccination group.

Dot blot assays were employed to evaluate the antigen-antibody reactivity of six peptides using sera collected from both unvaccinated and vaccinated individuals, in order to assess the production of IgG antibodies against the antigenic epitopes of the peptides in vaccinated individuals. The results demonstrated that the antibodies bound to at least one of the peptides P2, P5, or P6, which were present in the sera of various vaccinated individuals (**Figure 3A**). Some vaccinated individuals produced IgG antibodies reactive to P6, in contrast to the group with confirmed COVID-19 (**Figure 3A**). In vaccinated group, sera containing antibodies that bound exclusively to P2 accounted for 55.6%; those exclusive to P6 accounted for 11.1%; and sera reactive only to P5 represented 2.2% (**Figure 3C**). Additionally, the proportions of sera containing antibodies capable of binding two or three peptides (P2, P5, and P6) were as follows: P2+P5 (11.1%), P2+P6 (11.1%), P5+P6 (2.2%), and P2+P5+P6 (6.7%) (**Figure 3C**). Overall, P2 exhibited the highest positive response rate (84.4%) in the vaccinated group, followed by P6 with a positivity rate of 31.1%, while P5 showed the lowest positivity rate (22.2%) (**Figure 3D**). In addition, antibodies reacting with P1, P3, and P4 were not detected in individuals who had received two doses of the inactivated vaccine. Weak reaction signals were observed when sera from some unvaccinated individuals were incubated with SARS-CoV-2 S protein; however, no reactivity was detected with any of the six peptides (P1–P6), suggesting that P2, P5, and P6 peptides may offer greater specificity than the S protein as reagents for detecting antibodies to SARS-CoV-2 (**Figure 3B**). These findings suggest significant individual variation in serum antibody responses and profiles after vaccination. Among the three reactive peptides, P2 showed the strongest binding capacity, followed by P6, while P5 demonstrated the weakest.

### 3.5 Multiple sequence alignment of peptides with SARS-CoV-2 variants

**Figure 4:**
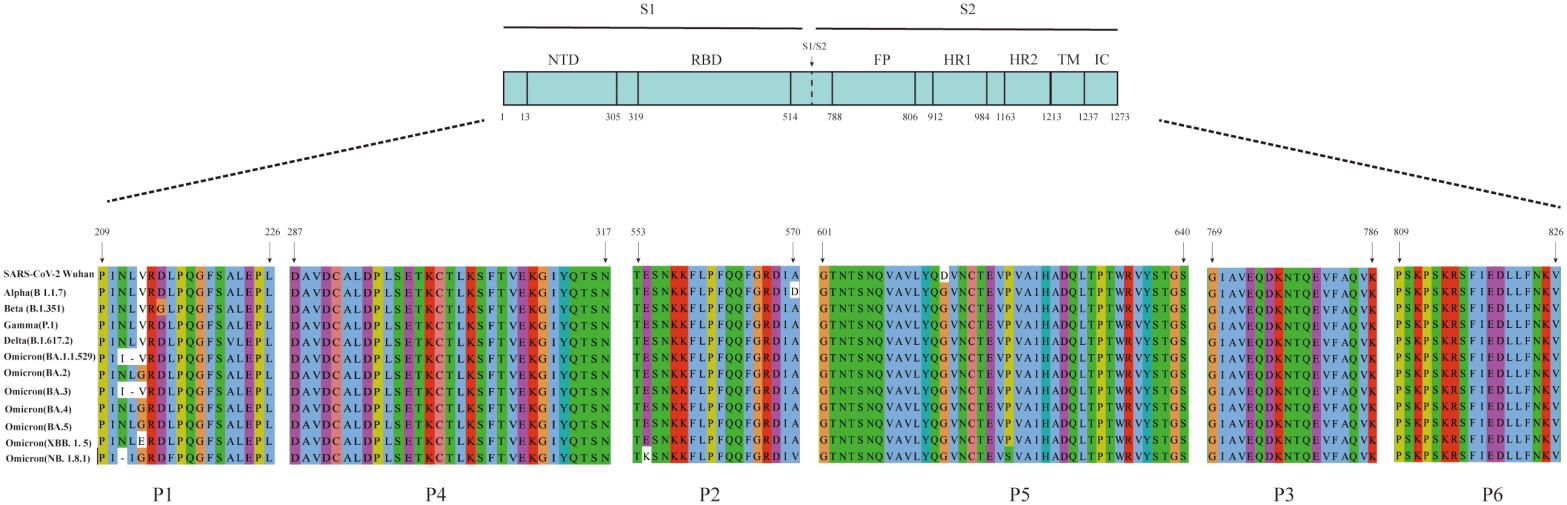
Amino acid sequence alignment of the six peptides with the corresponding regions of SARS-CoV-2 variants. Multiple sequence alignment of the six peptides with the corresponding regions of the S protein from SARS-CoV-2 variants, including Alpha (B.1.1.7), Beta (B.1.351), Gamma (P.1), Delta (B.1.617.2), Omicron (BA.1.1.529), Omicron (BA.2), Omicron (BA.3), Omicron (BA.4), Omicron (BA.5), Omicron (XBB.1.5), and Omicron (NB.1.8.1).

The amino acid sequences of the six B-cell linear peptides were compared with corresponding regions in the Alpha, Beta, Gamma, Delta, and Omicron subvariants. P1, P2, and P5 showed amino acid mutations in different variants: positions 211, 212, and 213 in P1 were mutated in several variants; P2 contained the A570D mutation in the Alpha strain and the E554K mutation in the NB.1.8.1 strain; and P5 had the D614G mutation in all epidemic variants (**Figure 4**). In contrast, P3, P4, and P6 remained highly conserved across all variants (**Figure 4**).

## 4 Discussion

In this study, six linear B-cell peptides from the S protein with immune advantages were screened using IEDB, and their reactivity with clinically collected serum samples was evaluated through dot blot assays. Although sera from patients with COVID-19 and vaccinated volunteers showed varying reactivity with the six peptides, P2 (aa 553–570), P5 (aa 601–640), and P6 (aa 809–826) exhibited strong IgG antibody reactivity in both groups. P2 showed the highest frequency and strongest antigen reactivity signal among the serum samples. Anti-P2 antibodies were detected in all patients during the early stage of SARS-CoV-2 infection, while anti-P5 antibodies appeared during the middle stage; antibodies against the other four peptides were not detected. However, anti-P6 antibodies were observed in some vaccine recipients, suggesting that individuals produce diverse antibody profiles after vaccination.

Variations in IgG antibody profiles among individuals have been reported for both patients with COVID-19 and vaccine recipients (Fraley et al., 2021; Li et al., 2021; Ma et al., 2021; Polvere et al., 2022). Polvere et al. found that antibodies to each peptide exhibited unique response characteristics in patients infected with SARS-CoV-2 and recipients of mRNA-based or DNA-based vaccines, and demonstrated that antibody profiles varied depending on the vaccine type or among individuals who received the same vaccine (Polvere et al., 2022). Fraley et al. analyzed serum antibody profiles following a single dose of the mRNA vaccine BNT162b2 and found that antibody hotspots recognizing the S protein region were primarily distributed in the S1-NTD (aa 139–150), RBD (aa 361–372), S1/S2 cleavage sites (aa 625–636) and (aa 661–672), aa 962–973 of heptad repeat 1, and aa 1148–1159 of heptad repeat 2 (Fraley et al., 2021). Using an S-protein peptide microarray, sera from individuals who received two doses of the inactivated virus vaccine BBIBP-CorV showed IgG antibodies derived from the sera of vaccinees that more readily recognized multiple peptide regions of the S protein, including aa 109–174 of S1-NTD, aa 331–384 of RBD, aa 902–937 of heptad repeat 1, and aa 1190–1219 of heptad repeat 2 (Ma et al., 2021). These findings indicate that the antibody profiles elicited by the mRNA vaccine BNT162b2 and the inactivated vaccine BBIBP-CorV demonstrate overlap within the S1-NTD (aa 139–150) and RBD (aa 361–372) regions, but exhibit distinct differences in other major antigenic sites.

Li et al. reported discovered that the linear epitopes bound by antibodies in the serum of patients with COVID-19 were predominantly located within the S1-CTD (aa 553–684) or near the S2’ cleavage site (aa 764–829) (Li et al., 2020). The major highly immunogenic epitopes identified included: S1-93 (aa 555–564), S1-97 (aa 577–588), S1-105 (aa 625–636), S1-106 (aa 631–642), S2-15 (aa 770–781), S2-22 (aa 812–823), and S2-23 (aa 818–829) (Li et al., 2021). The peptides P2 (aa 553–570), P5 (aa 601–640), and P6 (aa 809–826) identified in this study are located within the S1-CTD region or the S2’ cleavage site region. The epitopes P2, P5, and P6 fully encompass previously reported immunodominant epitopes (S1-93, S1-105, S1-106, S2-22, and S2-23) and exhibit extended amino acid lengths (Fraley et al., 2021; Li et al., 2021). Specifically, P2 spans a broader amino acid region than S1-93; P5 not only encompasses the combined region of S1–105 and S1–106 but also extends into additional sequences; and P6 covers a longer region than the synthesis of S2–22 and S2-23.

In this study, the positive rate of the antigen–antibody reaction for P2 (84.4%) was the highest among the vaccinated sera. Moreover, antibodies against P2 were detected in all infected sera. As reported by Li et al., the natural infection sera exhibited a relatively high positive rate for S1-93 (41.4%), indicating that P2 is a major antigenic epitope and a target site for antibody binding (Li et al., 2021). Interestingly, antibodies against P5 were detected in two out of the four cases in this study, whereas no antibodies targeting P6 were detected. Li et al. reported that the seropositivity rates for S1–105 and S1–106 in naturally infected individuals were 45.2% and 40.3%, while those of S2–22 and S2–23 were 20.2% and 3.9%, respectively (Li et al., 2021). Therefore, the antibody-positive rate of P5 (50.0%) observed in the serum from infected individuals in this study was slightly higher than that reported for S1–105 and S1–106 by Li et al (Li et al., 2021).

This suggests that the extended region of P5 beyond the S1-105 + S1–106 combined sequence is not redundant and may enhance the detection rate of antibody positivity. Subsequently, samples from additional COVID-19 patients will be collected for validation. In this study, the positive rate of the P5 antibody in sera from inactivated vaccine recipients was 22.2%, which was lower than that of the S1-105 (64.3%) and S1-106 (28.6%) antibodies in sera from mRNA vaccine recipients (Fraley et al., 2021). In contrast, the positive rate of the P6 antibody (31.1%) was higher than that of the S2-22 (21.4%) and S2-23 (21.4%) antibodies in sera from mRNA vaccine recipients (Fraley et al., 2021). Similarly, compared with the S1-105/S1–106 and S2-22/S2–23 antibodies in patients with COVID-19, the positive rate of the P5 antibody in sera from inactivated vaccine recipients was lower, while that of the P6 antibody was higher. Furthermore, similar to P5, the extended sequence of P6 beyond the S2-22 + S2–23 combined region is not redundant and may also contribute to an increased antibody detection rate. The positive rates of S1-105, S1-106, S2-22, and S2–23 differed between patients with COVID-19 and mRNA vaccine recipients (Fraley et al., 2021; Li et al., 2021). These findings suggest that differences exist in the antibody response profiles between vaccinated individuals and those with natural infection, which is consistent with the report by Polvere et al (Polvere et al., 2021). However, whether differences in the antibody profiles are attributed to vaccination or natural infection requires further investigation.

We identified three immunodominant linear B-cell epitopes (P2, P5, and P6), all of which are located on the surface of the S protein, which facilitates antibody binding due to their spatial accessibility. Although all three peptides are located on the surface of the S protein, the lower reactivity of P5 and P6 compared to P2 in vaccinated individuals may be due to partial shielding by the trimeric spatial conformation of the S protein, which could hinder antibody recognition (Li et al., 2020, 2021). The BNT162b2 (mRNA-based) vaccines elicited anti-RBD IgG antibodies in healthy individuals, with levels higher than those observed in convalescent patients (Polvere et al., 2022). Moreover, Nitahara et al. analyzed antibody epitope profiles in individuals vaccinated with the Pfizer BioNTech COVID-19 mRNA vaccine and found that the linear antibody responses against RBD were more diverse than those in naturally infected individuals (Nitahara et al., 2021). In our study, antibodies against P2 and P5 were observed in the serum of COVID-19 patients, while antibodies against three peptides (P2, P5, and P6) and their combinations were detected in the serum of individuals vaccinated with BBIBP-CorV. These findings suggest that the antibody profiles elicited vary among different individuals. Whether the types and combinations of antibodies produced in the serum of vaccine BBIBP-CorV recipients are more diverse than those in patients with COVID-19 requires confirmation with a larger sample size.

The six peptides reacted with commercial rabbit polyclonal antibodies to the S protein of SARS-CoV-2 and SARS-CoV, respectively. Among them, only P6 showed a strong and specific reaction with the anti-SARS-CoV-2 S protein antibody. Peptides P1, P2, and P5 showed immunological reactions with both anti-SARS-CoV-2 and anti-SARS-CoV S protein antibodies, likely due to the high homology between the two viruses in these peptide regions (**Supplementary Tables 2**). However, no antigen–antibody reactions were observed between peptides P1 and P6 and sera from patients with confirmed COVID-19. This discrepancy may reflect differences between the commercial antibody reagents, derived from recombinant S protein-immunized rabbits, and the sera of individuals naturally infected with SARS-CoV-2.

Serological testing plays a key role in the diagnosis and outbreak surveillance of COVID-19. Most commercial COVID-19 serological antibody diagnostic kits use full-length proteins or subunits such as the S protein, RBD region, and N protein as antigens. These antigens may cross-react with sera from individuals previously infected with other HCoVs (Lou et al., 2020; Zhao et al., 2020; Hicks et al., 2021). Ongoing mutations also introduce uncertainty in antigen selection during the transmission of SARS-CoV-2 (Lorenzo et al., 2021). Compared with full-length proteins, linear peptides offer advantages such as ease of preparation, high purity, and low cost, which make them attractive targets for serological analysis (Skwarczynski and Toth, 2016). Linear B-cell peptides from SARS-CoV-2 structural proteins have been predicted using phage display libraries (Guo et al., 2021), overlapping peptide pools (Poh et al., 2020), peptide arrays (Lorenzo et al., 2021) and bioinformatics prediction (Jespersen et al., 2017), allowing the identification of valuable immunogenic peptides for COVID-19 diagnosis and treatment. Peptides P1, P3, and P4 partially overlapped with peptide chain regions reported in previous studies but showed lower reactivity in sera from patients with confirmed COVID-19 compared with peptides P2, P5, and P6 (Amrun et al., 2020; Li et al., 2021; Polvere et al., 2021).

Different SARS-CoV-2 variants continue to undergo recombination and mutation during transmission. The World Health Organization classifies variants with increased transmissibility or virulence as variants of concern (Suzuki et al., 2022). The Omicron variant continues to circulate globally, with the NB.1.8.1 strain currently emerging as the predominant strain (Uriu et al., 2025). A multiple sequence alignment was performed comparing the amino acid sequences of peptides P2, P5, and P6 with those of circulating strains across different periods. In this analysis, P2 had an A570D mutation in the Alpha (B 1.1.7) variant and an E554K mutation in the Omicron (NB.1.8.1) variant. The D614G mutation was consistently present in P5 across all compared strains, whereas P6 remained highly conserved. The D614G mutation in the variants has been associated with increased transmissibility, greater adaptability, and higher viral load (Hou et al., 2020; Volz et al., 2021). Moreover, the combination of A570D and D614G mutations in the Alpha (B 1.1.7) variant has been shown to promote conformational changes in the RBD, thereby enhancing viral infectivity (Yang et al., 2021). NB.1.8.1, a descendant lineage of JN1, carries the E554K mutation, which may facilitate immune evasion by interfering with neutralization by monoclonal antibodies targeting the SD1 region of CTD (Afzal et al., 2025; Guo et al., 2025). In addition to SARS-CoV-2, six other HCoVs are known to cause disease, including four associated with the common cold (HCoV-229E, -HKU1, -NL63, and -OC43), as well as MERS-CoV and SARS-CoV. Multiple sequence alignment of the amino acid sequences showed that the S2 subunit and N protein exhibit high sequence homology between SARS-CoV-2 and these other HCoVs (Li and Li, 2021). Furthermore, these conserved regions were shown to exhibit cross-reactivity with serum IgG antibody from patients with COVID-19 (Camerini et al., 2021). By contrast, the S1 subunit of SARS-CoV-2 shows lower homology with the other six HCoVs. Notably, even the S1 subunit of SARS-CoV, while more similar, exhibits a very low frequency of cross-reactivity with sera from patients with confirmed COVID-19 (Okba et al., 2020). These findings suggest that the three immunodominant peptides described above have potential as target antigens for serological diagnosis of SARS-CoV-2 and provide an experimental basis for the development of peptide-based serological antibody diagnostic reagents.

In the unvaccinated group, the S protein showed weak spot reactions with the serum IgG antibodies of some individuals, whereas peptides P2, P5, and P6 showed no immunoreactivity, suggesting that the peptides are more specific than the S protein. Owing to individual differences in immune response, the detection rate of IgG antibodies against each peptide varies greatly. A combination of different peptides may enhance the overall positive detection rate and serve as a method for monitoring antibody protection after vaccination (Polvere et al., 2021). Whether the combination of P2, P5, and P6 has diagnostic and monitoring potential after vaccination requires validation through large-scale sampling and neutralization testing in future studies.

## Data Availability

The datasets presented in this study can be found in online repositories. The names of the repository/repositories and accession number(s) can be found in the article/**Supplementary Material**.
