# Supplementary material for: Identification of dominant peptide epitopes and antibody response to the spike protein of SARS-CoV-2
Source: Front Cell Infect Microbiol. 2025 Oct 15;15:1670219. doi: 10.3389/fcimb.2025.1670219 (PMC12568650; doi:10.3389/fcimb.2025.1670219)
Supplement: Supplementary file 1 [file Table1.docx]

**Supplementary Table 1. Characteristics of the study participants.**

|  | Vaccinated group  (n=45) | Unvaccinated group  (n=20) | Confirmed COVID-19 group  (n=4) |
| --- | --- | --- | --- |
| General characteristics |  |  |  |
| Age (years) | 33(23.0, 42.5) | 46(27.3, 56.8) | 46(31.8, 54.3) |
| Sex, n(%) |  |  |  |
| Male | 17(37.8) | 9(45.0) | 4(100.0) |
| Female | 28(62.2) | 11(55.0) | 0(0) |
| Comorbidities, n(%) |  |  |  |
| Diabetes | 2(4.4) | 1(5.0) | 2(50.0) |
| Hypertension | 1(2.2) | 2(10.0) | 1(25.0) |
| Cardiovascular disease | 1(2.2) | 3(15.0) | 1(25.0) |

Values are expressed as median (Q1, Q3) or n (%)

Supplementary Table 2. Comparison of six B-cell peptides identities on the spike protein between SARS-CoV-2 and SARS-CoV

| SARS-CoV-2 | | | | SARS-CoV | | |
| --- | --- | --- | --- | --- | --- | --- |
| Peptide No. | Sequence | Starting and ending amino acid |  | Sequence | Starting and ending amino acid | Identity (%) |
| 1 | PINLVRDLPQGFSALEPL | 209–226 |  | PIDVVRDLPSGFNTLKPI | 202–219 | 61 |
| 2 | TESNKKFLPFQQFGRDIA | 553–570 |  | TPSSKRFQPFQQFGRDVS | 539–556 | 67 |
| 3 | GIAVEQDKNTQEVFAQVK | 769–786 |  | GIAAEQDRNTREVFAQVK | 751–768 | 83 |
| 4 | DAVDCALDPLSETKCTLKS  FTVEKGIYQTSN | 287–317 |  | DAVDCSQNPLAELKCSVKS  FEIDKGIYQTSNF | 274–304 | 68 |
| 5 | GTNTSNQVAVLYQDVNCT  EVPVAIHADQLTPTWRVYSTGS | 601–640 |  | GTNASSEVAVLYQDVNCT  DVSTAIHADQLTPAWRIYSTGN | 587–626 | 78 |
| 6 | PSKPSKRSFIEDLLFNKV | 809–826 |  | PLKPTKRSFIEDLLFNKV | 791–808 | 89 |

Sequences were obtained from GenBank (accession numbers: NC_045512.2 and NC_004718.3)
